# Discovery and Characterization of a Birnavirus Causing Mortality in Cultured Silver Perch (*Bidyanus bidyanus*)

**DOI:** 10.1155/tbed/6843865

**Published:** 2026-11-15

**Authors:** Yuchao Gu, Hongyu Yan, Yan Liu, Xiaoru Zhang, Naiyou Zhang, Shumei Liu, Hongjing Zhang, Shaoping Weng, Jianguo He, Jian He

**Affiliations:** ^1^ State Key Laboratory for Biocontrol Southern Laboratory of Ocean Science and Engineering Guangdong (Zhuhai) School of Marine Sciences Sun Yat-Sen University Zhuhai China sysu.edu.cn; ^2^ School of Life Sciences Sun Yat-Sen University Guangzhou China sysu.edu.cn; ^3^ Guangdong Province Key Laboratory for Aquatic Economic Animals Sun Yat-sen University Guangzhou China sysu.edu.cn

**Keywords:** *Bidyanus bidyanus*, birnavirus, metatranscriptomics, viral disease

## Abstract

In July 2024, a disease outbreak occurred among cultured silver perch (*Bidyanus bidyanus*) in Guangzhou, Guangdong Province, China. Affected fish exhibited skin darkening and uncoordinated swimming, with lateral rolling in severe cases. Through metatranscriptomic analysis, it was found that Birnaviridae might be the potential pathogen of this disease and a birnavirus strain *Bidyanus bidyanus* birnavirus (BBBV), was successfully isolated from diseased silver perch using the SSN‐1 clone E‐11 cell line. Transmission electron microscopy (TEM) revealed that the viral particles were nonenveloped and single‐shelled, with a diameter of about 60 nm. Genome sequencing demonstrated that BBBV possesses a bipartite genome, consisting of segment A (3450 bp) and segment B (2657 bp). Phylogenetic analysis based on RNA‐dependent RNA polymerase (RdRp) sequences showed that BBBV clustered within the same clade as largemouth bass birnavirus (LBBV) and mandarin fish birnavirus (MFBV), with amino acid identities of 98.36% and 98.13% in RdRp and 95.69% and 95.87% in VP2, respectively. Experimental challenge resulted in higher mortality in BBBV‐infected fish than in the control group, with cumulative mortality rates of 70%, 100%, and 100% at 10^5^, 10^6^, and 10^7^ TCID_50_/mL, respectively. The virus was successfully reisolated from infected individuals, thereby fulfilling Koch’s postulates. Collectively, these findings demonstrate that BBBV is the causative agent of this disease in cultured silver perch, representing the first report of viral disease in this species and indicating a potential threat to silver perch aquaculture in China.

## 1. Introduction

Birnaviridae are nonenveloped viruses with a double‐stranded RNA genome divided into two segments [1]. Segment A encodes a large polyprotein that is cleaved to produce the structural proteins VP2 and VP3 together with the viral protease VP4, while segment B encodes VP1, an RNA‐dependent RNA‐polymerase responsible for viral genome replication [2, 3]. The family Birnaviridae comprises seven genera: *Aquabirnavirus*, *Avibirnavirus*, *Blosnavirus*, *Entomobirnavirus*, *Dronavirus*, *Telnavirus*, *and Ronavirus*. Among them, *Aquabirnavirus*, *Blosnavirus*, *Telnavirus*, and *Ronavirus* can infect aquatic organisms [1]. Birnaviruses are important viral pathogens in aquaculture. The infectious pancreatic necrosis virus (IPNV) is an important pathogen of fish that can cause substantial economic losses in aquaculture due to high morbidity and mortality [4]. Tellina virus 1 (TV‐1) was isolated from the marine bivalve mollusk *Tellina tenuis* and belongs to *Telnavirus* [5]. Blotched snakehead virus (BSNV), a member of the genus *Blosnavirus*, was originally isolated from a cell line derived from blotched snakehead (*Channa lucius*) [6]. In recent years, additional fish‐associated members of this family have been identified. Largemouth bass birnavirus (LBBV) was identified from diseased largemouth bass (*Micropterus salmoides*) and was associated with high mortality in aquaculture populations [7]. *Lates calcarifer* birnavirus (LCBV) was detected in Asian seabass (*Lates calcarifer*) and was linked to relatively mild clinical signs [8]. Mandarin fish birnavirus (MFBV) was isolated from diseased mandarin fish (*Siniperca chuatsi*) and was reported to cause acute infection with considerable mortality in juvenile fish [9].

The silver perch (*Bidyanus bidyanus*) is a freshwater fish belonging to the order Perciformes, family Terapontidae, and genus *Bidyanus* [10]. Native to the Murray–Darling Basin of Australia, this species has attracted increasing attention in aquaculture because of its rapid growth, omnivorous feeding habits, and high‐quality flesh rich in omega fatty acids [11]. Silver perch also exhibits strong environmental adaptability and can tolerate a wide temperature range from 2 to 38°C, which has facilitated its introduction into aquaculture in several countries, including China, Israel, and the Philippines [12]. Silver perch are susceptible to a range of infectious diseases. Parasitic infections are frequently reported and are mainly associated with pathogens, such as *Ichthyophthirius multifiliis* (causes the disease ichthyophthiriosis or white spot), *Chilodonella hexasticha* (chilodonellosis), *Trichodina* spp. (trichodinosis), and *Ichthyobodo necator* (ichthyobodosis) [12]. Fungal pathogens, particularly *Saprolegnia parasitica* and *Aphanomyces invadans*, can also cause severe disease outbreaks under unfavorable environmental conditions [12, 13]. In addition, bacterial pathogens, including *Flexibacter columnaris* and *Aeromonas salmonicida nova*, have been reported to infect silver perch and cause disease [12, 14]. Importantly, to date, no cases of viral diseases have been documented in cultured silver perch.

In July 2024, a disease outbreak occurred in cultured silver perch in Guangzhou, Guangdong Province, China, resulting in substantial economic losses. Through metatranscriptomic analysis combined with experimental infection and virus reisolation, a birnavirus strain *Bidyanus bidyanus* birnavirus (BBBV) was identified as the causative agent of the outbreak. This study represents the first report of a viral disease in cultured silver perch, highlighting a potential pathogenic threat and providing a basis for disease prevention and control strategies.

## 2. Materials and Methods

### 2.1. Sample Collection and E‐11 Cell Culture

In July 2024, naturally diseased and healthy silver perch samples were collected from aquaculture ponds at the breeding base of Guangzhou Haofeng Fishery Development Co., Ltd. in Guangzhou City, Guangdong Province. The fish were approximately 1 month old and 2–5 cm in body length. The affected ponds were managed separately from ponds containing healthy fish, and diseased fish were not co‐housed with healthy fish. Tissue samples including the intestine and visceral mass (a mixture of liver, spleen, and kidney) were aseptically collected. A portion of each tissue sample (<0.5 cm^3^) was immediately immersed in RNA later (Invitrogen, USA) to preserve RNA integrity and transported to the laboratory on dry ice, where it was stored at −80°C. Another subset of tissues was fixed in either 4% paraformaldehyde (for histopathological analysis) or 2.5% glutaraldehyde (for electron microscopy observation), with tissue thickness strictly controlled within 2 mm, then transported on ice packs and stored at 4°C. The remaining tissues were directly placed in sterile cryotubes, transported on dry ice, and stored at −80°C until further processing. The E‐11 cell line, a clone derived from the SSN‐1 fish cell line and originally established from striped snakehead, has been extensively used for the isolation and culture of viral strains [15, 16]. In this study, E‐11 cells were kindly provided by Shenzhen Customs and maintained in Leibovitz’s L‐15 medium supplemented with 10% fetal bovine serum (FBS) at 25°C. Cells at passage 4 (P4) were used for BBBV isolation and propagation.

### 2.2. RNA‐Seq Library Preparation, Sequencing, and Metatranscriptome Assembly

For metatranscriptomic analysis, tissue samples transported on dry ice were thawed on ice, rinsed with sterile phosphate‐buffered saline (PBS, pH 7.4), and homogenized in PBS using a sterile glass homogenizer. Total RNA was extracted using TRIzol Reagent (Invitrogen, USA) according to the manufacturer’s instructions. Sequencing libraries were prepared using the VAHTS Universal V8 RNA‐seq Library Prep Kit for Illumina and sequenced on an Illumina NovaSeq PE150 platform (Guangzhou Yiyue Biotechnology Co., Ltd.). Quality‐controlled non‐rRNA reads were assembled de novo using MEGAHIT to generate contigs [17]. These contigs were searched against the NCBI viral reference database using BLASTn and the NCBI nonredundant (NR) protein database using DIAMOND BLASTx to identify potential viral sequences [18]. Candidate viral sequences were further aligned to the NCBI NR nucleotide (nt) database using BLASTn to remove sequences likely derived from host or bacterial genomes.

### 2.3. Virus Isolation

The birnavirus strain from the silver perch used for whole‐genome sequencing and subsequent in vitro and in vivo experiments was originally isolated in 2024 from the diseased silver perch described above, collected in Guangzhou, Guangdong Province, China. Visceral tissues were collected from each of three individual fish and ground separately, with nine volumes of sterile PBS (pH 7.4) added to each sample for homogenization. Each homogenate was centrifuged at 7500 × *g* for 10 min at 4°C, and the supernatant was filtered through a 0.22 μm membrane. For virus isolation, 300 μL of each filtrate was diluted in L‐15 medium supplemented with 10% FBS and used to infect E‐11 cells for 7 days. After three rounds of blind passage, infected cultures were subjected to repeated freeze–thaw cycles, followed by centrifugation at 5000 × *g* for 30 min at 4°C [19]. The clarified supernatant was subsequently filtered through a 0.22 μm sterile membrane.

### 2.4. Virion Purification

The birnavirus strain from the silver perch isolated after three passages in E‐11 cells was subjected to repeated freeze–thaw cycles, followed by centrifugation and filtration. The resulting virus preparation was then ultracentrifuged at 28,000 rpm for 1 h at 4°C using an Optima XE‐100 ultracentrifuge equipped with a Type 70 Ti fixed‐angle rotor (Beckman Coulter, USA). The pellet was resuspended in PBS and loaded onto a discontinuous sucrose density gradient (20%, 30%, 40%, 50%, and 60%, from top to bottom). Ultracentrifugation was performed at 200,000 × *g* for 1 h at 4°C using an SW 41 Ti rotor. The resulting white precipitates were resuspended in PBS. Transmission electron microscopy (TEM) revealed that the fraction located between the 30% and 40% sucrose layers contained the highest abundance of viral particles.

### 2.5. TEM

Intestinal tissues collected from naturally diseased silver perch and BBBV‐infected E‐11 cells were processed for TEM. The intestinal tissues were immediately fixed in 2.5% glutaraldehyde prepared in 0.1 M phosphate buffer (pH 7.4) for 24 h at 4°C and subsequently post‐fixed in 1% osmium tetroxide in the same buffer for 1 h. BBBV‐infected E‐11 cells were fixed sequentially in 2.5% glutaraldehyde and 2.0% osmium tetroxide, both prepared in 0.1 M PBS. Following fixation, the samples were dehydrated through a graded ethanol series, embedded in epoxy resin, and sectioned into ultrathin sections. The sections were stained with uranyl acetate and lead citrate and examined using a Philips CM10 transmission electron microscope.

### 2.6. Virome Sequencing and Genome Assembly

Purified virions were submitted to Annoroad Gene Technology (Beijing, China) for virome library construction, quality assessment, and sequencing on the Illumina NovaSeq PE150 platform. Raw sequencing reads were processed to remove adapters and low‐quality sequences, and the remaining reads were assembled de novo into contigs using SPAdes [20]. Contigs with high sequencing depth were compared against the NCBI NT database using BLASTn, and viral genome sequences were selected from each assembly. The results were then polished using Pilon to obtain the final viral genome sequence [21]. The nt sequences of BBBV genome segments A and B have been deposited in GenBank under accession numbers PZ833385 and PZ833386, respectively.

### 2.7. Genome Functions, Structure Prediction, and Phylogenetic Tree Construction

Protein‐coding genes in the viral genome were predicted using ORFfinder. The predicted protein sequences were compared with the NCBI NR database using DIAMOND [18], and functional annotations were assigned based on the best‐hit results with an E‐value cutoff of 1 × 10^−6^. Conserved proteins, such as the RNA‐dependent RNA polymerase (RdRp), were aligned using multiple sequence alignment. Multiple sequence alignments were generated using MAFFT [22], followed by trimming of poorly aligned terminal regions with trimAl [23]. The curated alignments were subsequently used for phylogenetic reconstruction with IQ‐TREE [24] under the LG substitution model with 1000 ultrafast bootstrap replicates.

### 2.8. Histopathology

Intestinal and visceral mass tissues from diseased fish were fixed in 4% paraformaldehyde for 24 h. The tissues were then submitted to Genecore Biotechnology Co., Ltd., Guangzhou, for histological sectioning. Tissue sections (3–4 μm) were prepared and stained with hematoxylin and eosin (H&E), followed by microscopic examination.

### 2.9. RNA Extraction and Reverse Transcription Quantitative PCR (RT‐qPCR) Analysis

Total RNA was extracted from cells using the SV Total RNA Isolation Kit (Promega, USA) and reverse‐transcribed into first‐strand cDNA with the Evo M‐MLV qPCR RT Kit (AG, China) according to the manufacturers’ instructions. Quantitative PCR was performed on a Roche LightCycler 480 system (Roche, Switzerland) using the SYBR Green Pro Taq HS Real‐Time qPCR Kit (AG, China). All reactions were run in triplicate under the following cycling conditions: 95°C for 1 min, followed by 40 cycles of 95°C for 15 s, 60°C for 15 s, and 72°C for 45 s. Gene expression levels of BBBV were normalized to the *β-actin* reference gene. Primer sequences are listed in Supporting Information 1: Table S1.

### 2.10. Experimental Challenge Tests

Healthy silver perch were obtained from a fish farm in Guangzhou, Guangdong Province, China, with a body length of approximately 2–5 cm and an average body weight of approximately 6 g. Before the experiment, all fish were acclimated in laboratory aquaria for 7 days under continuous aeration, with approximately 30% of the rearing water replaced daily. The water temperature was maintained at 26°C, the pH at 7.3–8.0, and dissolved oxygen (DO) above 5 mg/L. Commercial feed was provided twice daily. The BBBV inoculum used for the challenge experiment was prepared following three successive passages of the virus in E‐11 cells, with P4 E‐11 cells being used for virus propagation. After three freeze–thaw cycles, the infected cells were centrifuged at 8000 × *g* for 30 min, and the supernatant was collected, and the resulting supernatant was collected and directly used for the challenge experiment. Eighty fish were randomly selected and divided into four groups, with 20 fish per group, and each group was maintained in a separate aquarium within a recirculating aquaculture system (RAS). The three experimental groups were intraperitoneally injected with 100 μL of BBBV inoculum at titers of 1 × 10^5^, 1 × 10^6^, and 1 × 10^7^ 50% tissue culture infectious doses (TCID_50_) /mL, respectively, while the mock control group received 100 μL of BBBV‐free E‐11 cell culture supernatant by the same route. Before injection, fish were anesthetized with 40 mg/L MS‐222 and subsequently subjected to an intraperitoneal injection. The aforementioned rearing conditions were maintained throughout the challenge experiment. Following the challenge, fish were monitored daily for clinical signs and mortality. Dead fish were collected, and tissue samples of the liver, spleen, kidney, and intestine were collected. The tissues were homogenized and filtered for virus re‐isolation in E‐11 cells. In addition, liver, spleen, kidney, and intestine tissues from diseased fish were collected for histopathological analysis.

## 3. Results

### 3.1. Clinical Signs and Histopathology

The naturally diseased silver perch were collected from aquaculture ponds at the breeding base. The fish were approximately 1 month old and 2–5 cm in body length. The affected ponds were managed separately from ponds containing healthy fish, and diseased fish were not co‐housed with healthy fish. Diseased silver perch exhibited darkened body coloration and abnormal swimming behavior, including circling near the water surface, darting rapidly, losing balance, and rolling frequently. In severe cases, fish overturned laterally, exposing the ventral surface, and eventually sank to the bottom after death. Upon dissection, a noticeable accumulation of yellowish fluid was present within the intestinal tract (Figure 1A). Kidney, intestine, liver, and spleen tissues were collected from both diseased and healthy silver perch for histopathological examination (Figure 1B). In healthy fish, the renal architecture was well preserved, whereas diseased fish exhibited pronounced renal lesions, characterized by degeneration and vacuolation of tubular epithelial cells, along with nuclear pyknosis (Figure 1B, kidney). The intestinal villi of healthy fish were well organized and structurally intact, while diseased fish showed vacuolar degeneration of columnar epithelial cells, along with degeneration and necrosis in the submucosa. In addition, the intercellular spaces between epithelial and muscle layers were widened, with a loosened and disorganized arrangement, and focal epithelial cell detachment was evident (Figure 1B, intestine). Hepatic cords in healthy fish were well organized and tightly arranged, whereas they appeared disorganized and loosely arranged in diseased individuals, accompanied by hepatocellular degeneration (Figure 1B, liver). In the spleen of healthy fish, the splenic structure was intact, whereas diseased fish exhibited extensive vacuolation of splenic cells and disorganization of splenic architecture (Figure 1B, spleen).

**Figure 1:**
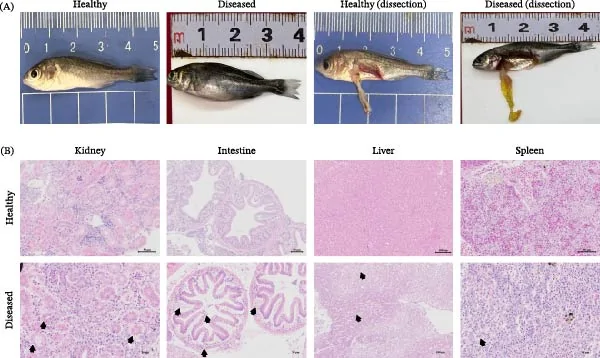
Clinical signs and histopathological changes in naturally diseased silver perch. (A) External appearance and anatomy of healthy and diseased silver perch. Diseased fish show darkened body coloration and accumulation of yellowish fluid in the intestinal tract. (B) The histopathology in different tissues of diseased silver perch. In the kidney, degeneration and vacuolation of tubular epithelial cells and nuclear pyknosis; in the intestine, vacuolar degeneration of columnar epithelial cells, submucosal degeneration and necrosis, and widening of the intercellular spaces between epithelial and muscle layers with a loose and disorganized arrangement; and in the liver, hepatocellular vacuolation; and in the spleen, extensive cellular vacuolation are observed (arrows).

### 3.2. Metatranscriptomic Analysis Using High‐Throughput Sequencing

Metatranscriptomic sequencing was conducted on pooled visceral tissues (liver, spleen, kidney, and intestine) from diseased silver perch. The assembled sequences were compared against the NCBI nt and nr databases, identifying two viral families: Birnaviridae and Adintoviridae. Phylogenetic analysis based on the RdRp of Birnaviridae (Figure 2A) and the hexon gene of Adintoviridae (Figure 2B) supported the presence of viral infection. All detected sequences were clustered within their corresponding family clades. In the phylogenetic tree, Birnaviridae‐related sequences are marked with red circles, whereas Adintoviridae‐related sequences are indicated by blue circles. Adintoviridae‐related sequences may represent viral‐derived elements that have integrated into host genomes. These fragments may exist in either complete or truncated forms within host DNA and typically lack infectious capacity [25, 26]. Birnaviruses are important pathogens in aquaculture and can cause substantial economic losses in fish farming [1]. These results imply that a birnavirus might be a potential pathogen of silver perch.

Figure 2Maximum‐likelihood phylogenetic trees based on the RNA‐dependent RNA polymerase of Birnaviridae and the hexon gene of Adintoviridae. (A) Maximum‐likelihood phylogenetic tree based on the deduced amino acid sequences of the RNA‐dependent RNA polymerase (RdRp) of Birnaviridae. The BBBV RdRp sequence was obtained from metatranscriptomic sequencing of diseased silver perch samples collected during the natural outbreak in Guangzhou, Guangdong Province, China. (B) Maximum‐likelihood phylogenetic tree based on the hexon gene of Adintoviridae. The Adintoviridae‐related hexon sequence was obtained from metatranscriptomic sequencing of silver perch samples collected during the same natural outbreak. Sequences related to Birnaviridae are indicated by red circles, whereas those related to Adintoviridae are marked with blue circles. The RdRp reference sequence used in this study was obtained from a previously published study [43]. Both trees were constructed using the maximum‐likelihood method with 1000 bootstrap replicates.

**Figure:**
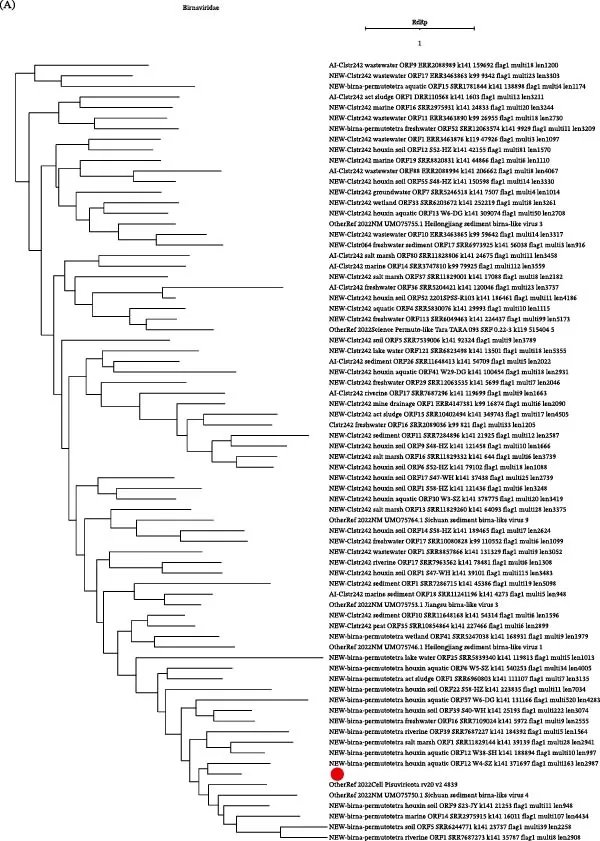

**Figure:**
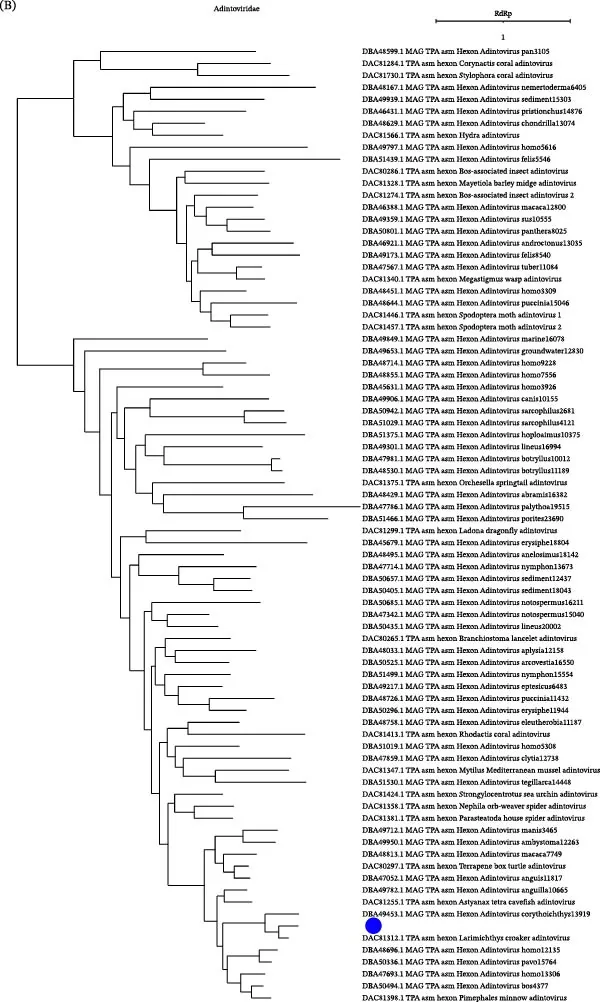

### 3.3. Virus Isolation From the Naturally Diseased Silver Perch

TEM examination of the intestinal tissue from diseased silver perch revealed viral particles approximately 60 nm in diameter (Figure 3A). TEM analysis of BBBV‐infected E‐11 cells further showed viral particles distributed in the cytoplasm near the cell membrane (Figure 3B). These viral particles were approximately 60 nm in diameter. In addition, viral particles were successfully purified by sucrose density gradient centrifugation, and their presence was further confirmed by TEM (Figure 3C). These results indicate that the virus was successfully isolated from silver perch. The filtered homogenate of mixed tissues was directly inoculated into E‐11 cells. Pronounced cytopathic effects (CPE) were observed at 48 h post‐infection with the third‐passage viral suspension, whereas control cells remained normal, as shown in Supporting Information 2: Figure S1.

**Figure 3:**
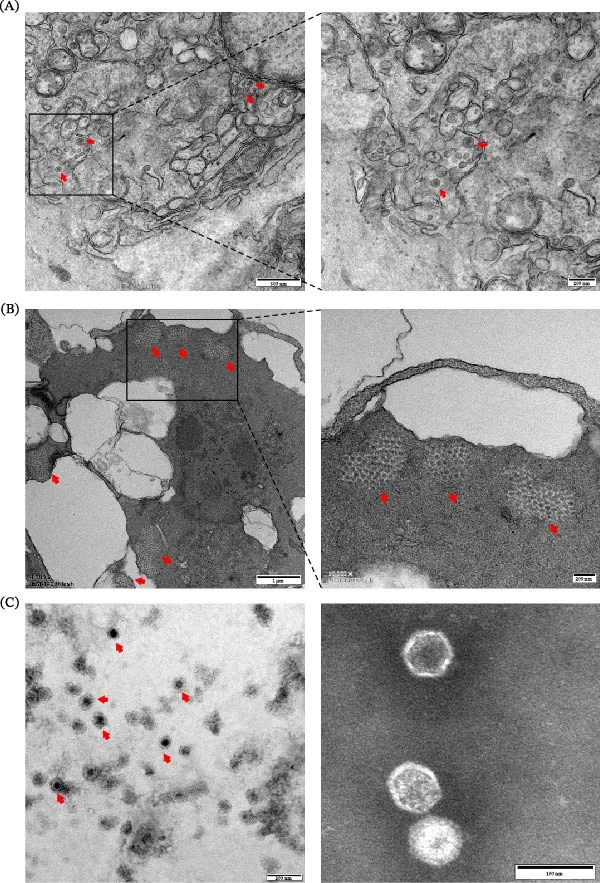
Virus isolation and characterization from diseased silver perch. (A) Transmission electron microscopy (TEM) image of intestinal tissue from a naturally diseased silver perch, showing viral particles (arrows). (B) Transmission electron microscopy of infected cells showing viral particles distributed in the cytoplasm near the cell membrane (arrows). (C) Negative‐stain electron micrograph showing the morphology and size of purified BBBV particles (arrows).

### 3.4. Whole‐Genome Characterization and Phylogenetic Analysis

BBBV genome was sequenced using next‐generation sequencing on the Illumina NovaSeq platform [27]. Genome assembly revealed that segment A was 3450 bp in length with a GC content of 56.41%. Segment A contains a single open reading frame (ORF) spanning nts 158–3427, encoding a polyprotein (pVP2–X–VP4–VP3), while segment B was 2657 bp in length with a GC content of 54.99% and contained an ORF spanning nts 83–2653, encoding the RdRp. The average sequencing depth was 13,806.4× for segment A and 18,693.7× for segment B, with genome coverage of 100% and 99.96%, respectively. The sequences were deposited in GenBank under accession numbers PZ833385 and PZ833386, respectively, as shown in Supporting Information 3: Figure S2. Comparative genomic analysis showed that, in contrast to IPNV (AJ622822.1 and AJ622823.1), BBBV encodes an additional polypeptide (designated X) located between the preVP2 and VP4 domains within the polyprotein of segment A [6, 28]. Notably, the nonstructural, positively charged polypeptide VP5, typically encoded by a small ORF in segment A of IPNV, was absent in the BBBV genome [28]. Furthermore, similar genomic features were observed in other related viruses, such as LBBV (MW727622.1 and MW727623.1) and MFBV (PP786692.1 and PP786693.1), which also contain the X protein but lack the VP5 coding sequence (Figure 4A). As the RdRp is the most conserved protein among RNA viruses, it is widely used for phylogenetic analysis [29]. A phylogenetic tree was constructed based on the RdRp amino acid sequences of 33 members of the Birnaviridae family. The results showed that BBBV, LBBV, and MFBV clustered into a distinct clade. Sequence identity analysis revealed that BBBV shared 98.36% and 98.13% amino acid identity with LBBV and MFBV, respectively (Figure 4B). The VP2 capsid protein is the major immunogenic protein of birnaviruses [30]. Similar clustering patterns were observed in the phylogenetic tree based on VP2 amino acid sequences, as shown in Supporting Information 4: Figure S3.

Figure 4Genomic organization and phylogenetic analysis of BBBV. (A) Schematic representation of the genome organization of BBBV and related viruses. Segment A encodes a polyprotein (pVP2–X–VP4–VP3), and segment B encodes the RNA‐dependent RNA polymerase. The additional X protein and the absence of VP5 are indicated in comparison with related viruses. (B) A phylogenetic tree of representative members of the Birnaviridae family was constructed based on the amino acid sequences of the RNA‐dependent RNA polymerase using the maximum likelihood method with 1000 bootstrap replicates. Branch lengths correspond to the number of inferred substitutions, as indicated by the scale bar. BBBV is highlighted with a red box in the tree. BBBV clusters with LBBV and MFBV, forming a distinct clade.

**Figure:**
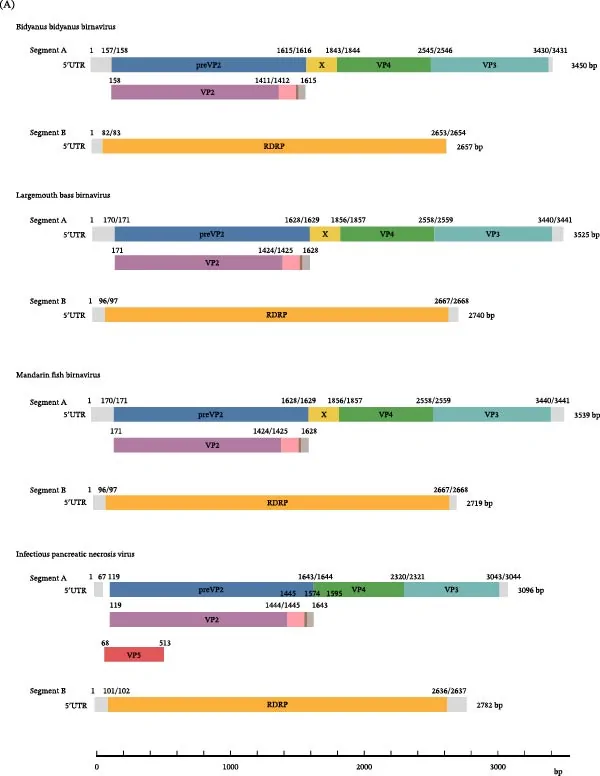

**Figure:**
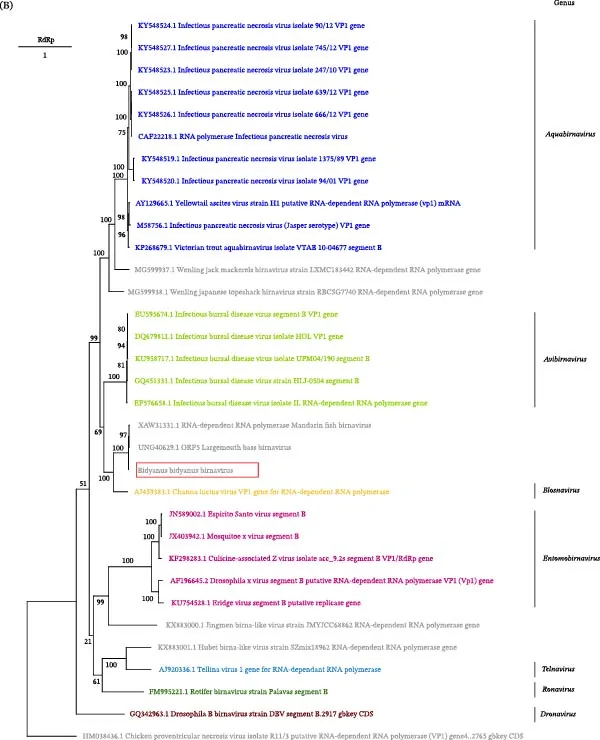

### 3.5. Experimental Infection and Pathogenicity Assessment

Mortality was observed in all three BBBV‐challenged groups, whereas no mortality occurred in the negative control group. The cumulative mortality rates were 70%, 100%, and 100% in fish challenged with BBBV at 1 × 10^5^, 1 × 10^6^, and 1 × 10^7^ TCID_50_/mL, respectively (Figure 5A). The relative BBBV RNA levels differed among tissues and sampling times following infection. BBBV RNA was detected in all examined tissues, with the highest levels generally observed in the kidney and intestine, particularly at 2 and 3 days post‐infection. The BBBV RNA level in the kidney increased markedly over time, whereas the intestine maintained relatively high levels at days 2 and 3. Significant temporal differences were observed in the spleen, kidney, intestine, and muscle. Relatively low BBBV RNA levels were detected in the liver, gill, and brain throughout the experiment (Figure 5B). These results fulfilled Koch’s postulates. Histopathological examination was performed on the kidney, intestine, liver, and spleen of diseased silver perch. In healthy fish, all examined tissues exhibited normal architecture, with no evident vacuolation, degeneration, or necrosis. In diseased fish, the kidney showed enlarged cells, along with degeneration and vacuolation in the proximal tubules and collecting ducts. The intestine exhibited separation of villi, degeneration and necrosis in the submucosa, and vacuolation with partial detachment of intestinal epithelial cells. The liver showed dilation and congestion of hepatic sinusoids, accompanied by hepatocellular vacuolation and lysis. The spleen exhibited marked vacuolation and cell lysis (Figure 5C). These results confirm that BBBV is the causative agent of disease in silver perch.

**Figure 5:**
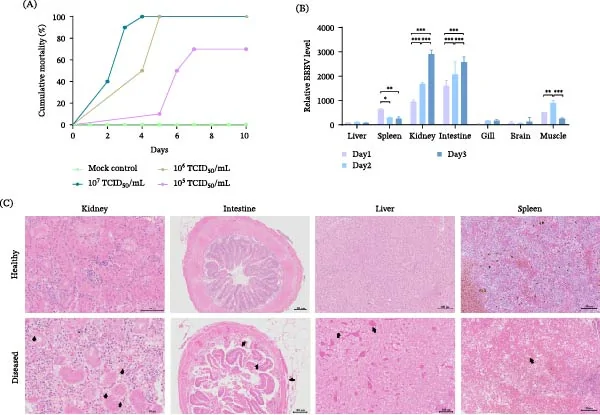
Challenge experiment, viral RNA quantification, and histopathological changes in experimentally challenged silver perch. (A) Cumulative mortality of silver perch challenged intraperitoneally with BBBV at doses of 1 × 10^5^, 1 × 10^6^, and 1 × 10^7^ tissue culture infectious doses (TCID_50_)/mL. The negative control group received BBBV‐free E‐11 cell culture supernatant. (B) Relative BBBV RNA levels in the liver, spleen, kidney, intestine, gill, brain, and muscle of experimentally infected silver perch were determined by RT‐qPCR at 1, 2 and 3 days post‐infection. Statistical significance between groups was determined by *t*‐test.  ^∗^ *p* < 0.05,  ^∗∗^ *p* < 0.01, and  ^∗∗∗^ *p* < 0.001. (C) Histopathological changes in kidney, intestine, liver, and spleen of experimentally challenged fish. In the kidney, enlarged cells and cellular vacuolation; in the intestine, villus separation, submucosal degeneration and necrosis, and epithelial cell detachment; in the liver, congestion of hepatic sinusoids and hepatocellular vacuolation; and in the spleen, cellular vacuolation are observed (arrows).

## 4. Discussion

In this study, metatranscriptomic sequencing was employed to screen and identify potential pathogens in diseased silver perch. Viral particles were subsequently isolated, propagated, and purified under laboratory conditions. Their pathogenicity was confirmed by challenge experiments, fulfilling Koch’s postulates [31]. These findings provide experimental evidence supporting the involvement of BBBV in the observed disease outbreak. This study represents the first report of a viral disease in cultured silver perch, characterized by obvious pale yellow fluid accumulation in the intestinal tract at necropsy, and indicates that BBBV was associated with the mass mortality observed during the silver perch farm outbreak.

According to the taxonomic framework defined by the International Committee on Taxonomy of Viruses (ICTV) for Birnaviridae, the classification of these viruses was assessed based on phylogenetic relationships and sequence similarity [1]. For taxa lacking clearly defined species demarcation criteria, a threshold of 90% amino acid sequence identity in the RdRp was adopted as the criterion for species delineation [32, 33]. Phylogenetic analysis based on RdRp sequences from different genera within the Birnaviridae revealed that BBBV does not belong to any recognized genus, but instead forms a monophyletic clade together with LBBV and MFBV. Sequence identity analysis further showed that BBBV shares 98.36% and 98.13% amino acid identity in the RdRp and 95.69% and 95.87% in the VP2 polyprotein, with LBBV and MFBV, respectively. Given that the amino acid identities of these key proteins all exceed the 90% threshold and that phylogenetic analysis places them within a distinct clade, we further propose that at the species level, these viruses may represent different strains of the same virus in three hosts. Meanwhile, BSNV formed a separate monophyletic lineage in the phylogenetic tree, whereas BBBV, LBBV, and MFBV were located in another lineage, with a bootstrap value of 100% (Figure 4B). In addition, the RdRp sequence similarity between BBBV and BSNV (NC_005983) did not exceed 69%. Previous studies have reported that the presence of the VP5 protein is genus‐specific [5], but no VP5 protein was detected in any of the three viruses. Taken together, we suggest that BBBV, LBBV, and MFBV may represent a distinct evolutionary lineage within Birnaviridae, which may warrant further investigation as a candidate novel genus.

Aquaculture viruses can be transmitted through multiple routes, such as waterborne transmission, movement of infected live aquatic animals, dietary transmission, direct contact between infected and susceptible hosts, vertical transmission through infected broodstock or reproductive products [34–36]. Among these routes, waterborne transmission is particularly important for aquatic birnaviruses [37]. For example, IPNV can remain infectious in freshwater and marine environments for extended periods, thereby increasing the risk of infection and potentially causing substantial economic losses in aquaculture [37, 38]. Silver perch was introduced into China in the 1980s. At present, silver perch in Guangdong Province is mainly cultured in outdoor earthen ponds. These aquaculture areas are also widely used for the culture of other economically important freshwater fish, including mandarin fish and largemouth bass. In this study, we identified a new birnavirus strain BBBV, which is the first report of viral pathogen in cultured silver perch. Interestingly, BBBV shares a high sequence similarity with MFBV and LBBV. Given the overlap in culture areas, farming practices, and water use among these fish species, cross‐species transmission may occur between them. Biological vectors also represent an important route of birnavirus transmission. For example, blue mussels (*Mytilus edulis*) can accumulate infectious IPNV from the surrounding water and may subsequently transmit the virus to cohabitating Atlantic salmon (*Salmo salar*) smolts, indicating that shellfish may serve as potential reservoirs or vectors of IPNV [39]. In these pond environments, mollusks, crustaceans, and other aquatic invertebrates may carry, accumulate, or mechanically transport viruses, thereby potentially facilitating viral persistence and dissemination as well as transmission between different cultured animals and water bodies [40–42]. Therefore, these intermediate hosts should not be neglected in the surveillance of silver perch, and ponds should be cleaned and disinfected before stocking to eliminate the intermediate hosts.

## 5. Conclusion

In summary, we report the first viral disease in cultured silver perch and describe the isolation and characterization of a birnavirus strain BBBV, with pathogenicity confirmed through challenge experiments. These findings expand the known host range of Birnaviridae and provide a basis for improving the diagnosis, surveillance, and control of viral diseases in silver perch. However, considering the limited sampling and geographic scope of this study, the distribution and transmission dynamics of BBBV remain to be further investigated. Future studies should focus on broader epidemiological surveillance and on elucidating the ecological interactions and transmission pathways of BBBV in aquaculture environments.

## Author Contributions

Conceptualization, funding acquisition, methodology, supervision, writing – review and editing: Jian He and Jianguo He. Data curation: Yuchao Gu and Hongyu Yan. Formal analysis, writing – original draft: Yuchao Gu and Jian He. Investigation: Yuchao Gu, Hongyu Yan, Yan Liu, Shumei Liu, Naiyou Zhang, Xiaoru Zhang, Hongjing Zhang, and Shaoping Weng.

## Funding

This work was supported by the National Key Research and Development Program of China (Grant 2023YFD2402900), the National Natural Science Foundation of China (Grant 32473128), and the Earmarked Fund for the China Agriculture Research System (Grant CARS‐46).

## Disclosure

All the authors have discussed the results, reviewed, and accepted the final contents of the manuscript.

## Ethics Statement

All animal experiments were approved by the Animal Ethics Committee of Sun Yat‐sen University (Approval Number SYSU‐IACUC‐2024‐B0286) and performed in strict compliance with the institution’s guidelines on animal welfare.

## Conflicts of Interest

The authors declare no conflicts of interest.

## Supporting Information

Additional supporting information can be found online in the Supporting Information section.

## Supporting information


**Supporting Information 1** Table S1: Primers used for real‐time RT‐qPCR. The table lists the forward and reverse primer sequences for BBBV and the reference gene β‐actin, with gene names and sequences (5′→3′) provided.

**Supporting Information 2** Figure S1: Cytopathic effects in E‐11 cells following inoculation with tissue homogenates from healthy and naturally diseased silver perch. (A) E‐11 cells before inoculation with tissue homogenate from a healthy silver perch. (B) E‐11 cells before inoculation with tissue homogenate from a naturally diseased silver perch. (C) E‐11 cells after inoculation with tissue homogenate from a healthy silver perch, showing no cytopathic effects. (D) E‐11 cells after inoculation with tissue homogenate from a naturally diseased silver perch, showing marked cytopathic effects. Scale bars = 100 μm.

**Supporting Information 3** Figure S2: Sequencing depth profiles of the BBBV genome. Coverage maps for segment A (3450 bp) and segment B (2657 bp). The *x*‐axis indicates the genome position (bp), and the *y*‐axis indicates the sequencing depth (×). The dashed line indicates the mean sequencing depth, and the values in the upper right corner indicate the percentage of the genome covered.

**Supporting Information 4** Figure S3: Phylogenetic analysis based on VP2 amino acid sequences. A phylogenetic tree of representative members of the Birnaviridae family was constructed based on the amino acid sequences of the VP2 using the maximum likelihood method with 1000 bootstrap replicates. Branch lengths correspond to the number of inferred substitutions, as indicated by the scale bar. BBBV is highlighted with a red box in the tree. The results showed that BBBV, largemouth bass birnavirus (LBBV), and mandarin fish birnavirus (MFBV) clustered into a distinct clade.

## Data Availability

The raw data supporting the conclusions of this article will be made available by the authors without undue reservation.
