# Supplementary material for: Discovery and Characterization of a Birnavirus Causing Mortality in Cultured Silver Perch (Bidyanus bidyanus)
Source: Transbound Emerg Dis. 2026 Nov 15;2026:6843865. doi: 10.1155/tbed/6843865 (PMC13770274; doi:10.1155/tbed/6843865)
Supplement: Supplementary file 1 [file TBED-2026-6843865-s001.docx]

**Supplementary Table S1 Primers for Realtime RT-qRCR**

| **Gene name** | **Sequence (5’–3’)** |
| --- | --- |
| ***BBBV-F*** | **AATGGCGGGCTTGACTCAATCAT** |
| ***BBBV-R*** | **TGCTGAACCAGATCCTGTCCTGA** |
| ***β-actin-F*** | **CCCTCTGAACCCCAAAGCCA** |
| ***β-actin-R*** | **CAGCCTGGATGGCAACGTACA** |
